# Roll-Your-Own Tobacco Use Among People Smoking Menthol Cigarettes in Great Britain, 2020-2023: A Population-Based Survey

**DOI:** 10.1093/ntr/ntae217

**Published:** 2024-09-10

**Authors:** Vera H Buss, Jamie Brown, Harry Tattan-Birch, Sarah E Jackson, Lion Shahab

**Affiliations:** Department of Behavioural Science and Health, University College London, London, UK; SPECTRUM Research Consortium, Edinburgh, UK; Department of Behavioural Science and Health, University College London, London, UK; SPECTRUM Research Consortium, Edinburgh, UK; Department of Behavioural Science and Health, University College London, London, UK; SPECTRUM Research Consortium, Edinburgh, UK; Department of Behavioural Science and Health, University College London, London, UK; SPECTRUM Research Consortium, Edinburgh, UK; Department of Behavioural Science and Health, University College London, London, UK; SPECTRUM Research Consortium, Edinburgh, UK

## Abstract

**Introduction:**

The sale of factory-made cigarettes with menthol as a characterizing flavor has been prohibited in Great Britain since May 2020. However, menthol accessories like flavored filters for roll-your-own (RYO) tobacco can be sold legally, possibly undermining the policy. This study aimed to explore the association between RYO and menthol cigarette smoking.

**Aims and Methods:**

Data were collected between October 2020 and October 2023 from a monthly population-based cross-sectional survey, with 82 120 adults (≥18) living in Great Britain providing complete data. Logistic regression models assessed the association between predominant RYO tobacco use and menthol cigarette smoking, and whether it differed by sociodemographic characteristics, unadjusted and adjusted for age, gender, ethnicity, nation, and socioeconomic position. Time trends in RYO tobacco use among people smoking menthol cigarettes were modeled over the study period.

**Results:**

There has been no clear decrease in menthol cigarette smoking prevalence among people who smoke (~14%) following the ban. Predominant RYO use increased among people smoking menthol cigarettes from 49.6% (95% CI: 42.2 to 57.0) in October 2020 to 61.9% (95% CI: 57.5 to 66.0) in June 2022, after which it remained stable. Predominant RYO use was more common among people smoking menthol than non-flavored cigarettes overall (adjusted odds ratio (OR_adj_) = 1.30, 95% CI: 1.14 to 1.49) and across demographic subgroups. This association was most pronounced in middle-aged compared with older people (35 vs. 65 years OR_adj_ = 1.18, 95%CI: 1.01 to 1.35), and in ethnic minorities compared with White people (OR_adj_ = 1.56, 95% CI: 1.03 to 2.36).

**Conclusions:**

There was a substantial increase in RYO use among people smoking menthol cigarettes in the first two years after the ban, from approximately 50% to 60%.

**Implications:**

The availability of menthol accessories may have undermined the ban on factory-made mentholated cigarettes in Great Britain. Roughly one in seven people who smoke cigarettes still report smoking menthol cigarettes and among these, about two-thirds predominantly use RYO tobacco. Since there has been no noteworthy change in the prevalence of menthol cigarette smoking since October 2020, new measures will likely be necessary to achieve a further reduction. For example, menthol accessories could be banned or their advertising and availability heavily restricted.

## Introduction

Menthol is the most common flavor added to cigarettes. It is particularly popular among youth (aged 12–17) and young adults (aged 18–24).^1,2^ Adding menthol can make tobacco less harsh to smoke,^1,2^ and some people mistakenly perceive menthol cigarettes to be less harmful than regular non-flavored cigarettes.^2,3^ Therefore, in May 2020, the European Union and the United Kingdom introduced a ban on characterizing flavors (that change the smell or taste of cigarettes), including menthol, in factory-made cigarettes and tobacco used in roll-your-own (RYO) cigarettes (more information about the ban is provided in Supplementary File).^4,5^ In 2016, prior to the menthol ban, Zatoński et al.^6^ reported that an estimated 12.4% of people who smoked in England usually smoked menthol cigarettes. Sales data from 2018 showed that menthol cigarettes, including those with menthol capsules, comprised 21% of the market share, up from 14% in 2014.^7^ The ban aimed to reduce smoking uptake among young people and to promote smoking cessation among those who previously smoked menthol cigarettes. However, evidence from studies conducted after the ban was implemented suggests that a substantial proportion of people who smoke in Great Britain still use menthol cigarettes.^8–11^ East et al.^9^ reported that, among people aged 16 to 19 years who smoked in England, 12.1% reported smoking menthol or capsule cigarette brands in February 2020, which decreased to 3.0% in August 2020. Kock et al.^10^ found that, in England between July 2020 and June 2022, a higher proportion of women and younger people who smoked reported menthol cigarette use than men and older people, but there was little difference by ethnicity. The present study aimed to assess whether the continued high prevalence of menthol cigarette smoking being reported among the British adult population may have been driven by the use of RYO cigarettes and menthol accessories.

People in Great Britain have several options available to them to still obtain menthol cigarettes. First, they could purchase illicit menthol cigarettes, but the evidence so far suggests that this option is not common.^8,10,12^ Second, people may buy commercially available cigarettes that they perceive to be mentholated, even though the manufacturers state that they do not contain characterizing flavors.^13–15^ Third, people may use accessories to add menthol flavor to their cigarettes. Such accessories are not covered by the ban and include mentholated filters, rolling paper, or spray for RYO tobacco, drops or crush balls that are added to the filter, or infusion cards that are placed in packs to infuse the cigarettes.^15,16^ There is reason to suspect that the combination of menthol accessories with RYO tobacco might be more popular than the combination with factory-made cigarettes because it might be more convenient, and the subjective experience might be closer to that of traditional menthol cigarettes. For example, crush balls require applicators to insert the ball in the cigarette filter and infusion cards must be placed in the packs about an hour prior to smoking, while menthol filters or papers can just be used instead of non-mentholated ones when rolling a cigarette, so no additional steps are required. Regarding the subjective experience, a study compared different menthol cigarette alternatives (RYO tobacco with menthol accessories, menthol-filtered little cigars or non-menthol cigarettes) and found the highest satisfaction among participants when using RYO tobacco with menthol accessories.^17,18^

The present study assessed whether RYO tobacco use was associated with menthol cigarette smoking among adults who smoked in Great Britain between October 2020 and October 2023. Furthermore, the study considered potential interactions between predominant RYO use and certain sociodemographic characteristics. For example, there could be differences by ethnicity as indicated by a recent study in which English youth from ethnic minority groups had disproportionately higher use of menthol cigarettes and menthol accessories than White youth.^11^ There may also be differences between men and women, with women generally more likely to smoke menthol cigarettes and in recent years increasingly reporting the use of RYO tobacco.^6,10,19^ This trend could be partially driven by women having switched from factory-made menthol cigarettes to RYO cigarettes with menthol accessories. The study also considered differences by age because marketing for menthol accessories, for example, via online shops, might have been more targeted toward younger people.^20^ Further, the three nations in Great Britain might differ from one another because the Scottish legislation prohibits the display of any smoking-related products in shops while the English and Welsh legislation only prohibit the display of tobacco.^21,22^ Hence, it is possible that people in Scotland are less aware of menthol accessories.

Additionally, the present study assessed trends over time in RYO tobacco use by menthol cigarette smoking, as people could have switched to RYO tobacco and menthol accessories in the first few months after the ban was introduced, or changes may have occurred over the subsequent years. Further, the study reported trends in the prevalence of menthol cigarette smoking among the total population, people who smoked, and by the type of cigarette they smoked (RYO vs. factory-made) to assess whether trends differed. Generally, there has been a steady increase in the proportion of people smoking predominantly RYO as opposed to factory-made cigarettes in England between 2008 and 2022.^19^

In summary, this study aimed to assess and characterize the prevalence of RYO cigarette use among people smoking menthol versus non-flavored cigarettes in Great Britain between October 2020 and October 2023. Specifically, we addressed the following research questions: (1) Overall, is predominant RYO use more prevalent among people who smoke menthol cigarettes compared with those who smoke non-flavored cigarettes? (2) Does any association between predominant RYO use and menthol use differ by ethnicity, gender, age, or nation? (3) To what extent has the prevalence of predominant RYO use by menthol cigarette smoking changed since October 2020? (4)To what extent has the prevalence of menthol cigarette smoking by predominant RYO use changed since October 2020?

## Methods

### Study Design

The study protocol including the analysis plan was preregistered on the Open Science Framework (https://osf.io/y8sge/). The University College London Ethics Committee granted ethical approval for the Smoking and Alcohol Toolkit Study (ID 0498/001). Data for this study were collected as part of the Smoking Toolkit Study, an ongoing monthly cross-sectional population-based survey. The Smoking Toolkit Study captures a range of data on smoking-related behaviors from a new sample of adults each month. Menthol cigarette use was first assessed in England in July 2020 (after the ban was implemented) and has been assessed monthly across all of Great Britain since October 2020. Because data on menthol cigarette use were not collected prior to the ban, it was not possible to examine changes from before to after the ban. Instead, we analyzed data collected between October 2020 (the first data collection in Scotland and Wales) and October 2023 from adults aged 18 years and older living in England, Scotland, or Wales. The sampling strategy consists of a combination of random location and quota sampling. A market research company conducted the interviews with survey participants via telephone. To ensure accessibility and inclusivity, participants could have someone with them who could assist with the call and further reasonable adjustments were made for participants with hearing impairments, such as slowing down and speaking as clearly as possible. The research team received anonymized data. More details on the survey methods are published elsewhere.^23,24^ The manuscript followed the STROBE (strengthening the reporting of observational studies in epidemiology) statement.^25^

### Outcome Variables and Covariates

All variables were self-reported. We determined cigarette smoking status with the following question: “Which of the following best applies to you?.” Participants who replied “I smoke cigarettes (including hand-rolled) every day” or “I smoke cigarettes (including hand-rolled), but not every day” were classified as cigarette smoking.

Those who reported cigarette smoking were subsequently asked: “Cigarettes can be sold in different flavors. They can also be flavored by capsules, filter tips, cards inserted into a packet, or flavored rolling papers. How would you describe the flavor of the cigarettes you usually smoke/smoked?.” If they replied “Tobacco and menthol,” they were classified as smoking menthol cigarettes, and if they replied “Just tobacco,” they were classified as smoking non-flavored cigarettes. Participants who answered “Tobacco and some other flavor” were excluded from the analysis.

People who smoked cigarettes were also asked how many cigarettes they usually smoked and how many of these were hand-rolled. We classified those who stated that at least 50% of their overall consumption are RYO cigarettes as predominantly smoking RYO cigarettes, and the remaining as predominantly smoking factory-made cigarettes. We have used the same cutoff in previous studies (eg, Jackson et al.^19,26^) and originally followed definitions used in a study by Young et al.^27^

Other variables used in the analysis included age, gender, ethnicity, nation, socioeconomic position, and time. Time was based on the survey wave and ranged from 1 (October 2020) to 37 (October 2023). We modeled age and time using restricted cubic splines with 3 knots (placed at the minimum, median, and maximum for age, and the beginning, middle, and end for time).^28,29^ Gender had three response options: woman, man, or non-binary. We included those who identified as non-binary in the overall sample but excluded them from the analysis by gender due to small numbers.

We grouped ethnicity into White or ethnic minorities. To explore differences between ethnic groups further (not preregistered), we categorized ethnicity into White (White British, White Irish, White Gypsy/Traveler, White other), Asian or mixed White/Asian (Asian Indian, Asian Pakistani, Asian Bangladeshi, Asian Chinese, Mixed White/Asian, Asian other), Black or mixed White/Black (Black African, Black Caribbean, mixed White/African Caribbean, mixed White/Black African, Black other), and other (Arab, other). This is based on the classification used in the 2021 Census of England and Wales^30^ with the only difference being that we did not include a separate group for mixed or multiple ethnic groups due to small sample sizes.

The variable for nation was divided into England, Scotland, or Wales. Socioeconomic position was based on the National Readership Survey’s classification of social grade^31^ and split into two categories: people with more advantaged social grade (ABC1: high and intermediate managerial, administrative, or professional, supervisory, clerical, and junior managerial, administrative or professional), and people with less advantaged social grade (C2DE: skilled manual workers, semi and unskilled manual workers, state pensioners, casual or lowest grade workers, unemployed with state benefits only).

### Analysis

We performed a complete case analysis, using RStudio (version 2022.07.2, R version 4.2.1). The number and percentage of missing values for each variable are listed in Table S1. Whenever the interviewer noted a participant's response as “Don’t know” or “Refused,” we assumed these to be missing. We weighted data using raking to match the population of Great Britain based on sociodemographic characteristics.^32^ Unweighted estimates are provided in Supplementary Material.

#### Associations Overall and by Subgroups (RQs 1–2)

We used a logistic regression model with predominant RYO use as the outcome and menthol cigarette smoking as the predictor. Then, in a series of models, we added an interaction term for whether someone smoked menthol cigarettes and each sociodemographic characteristic (menthol smoking by ethnicity, gender, age, or nation). For each model, we calculated unadjusted and adjusted odds ratios and their 95% confidence intervals (CIs). Covariates for adjustment included age, gender, ethnicity, social grade, and nation, as it is known that these characteristics are associated with RYO use among people who smoke in Great Britain.^19,26,33^

In addition to these preregistered analyses, we reported the prevalence of predominant RYO use among each subgroup, stratified by whether they smoked menthol or non-flavored cigarettes. Where there were significant interactions between menthol cigarette smoking and sociodemographic characteristics, we also reported (i) the adjusted odds of predominant RYO use among people smoking menthol cigarettes and (ii) the adjusted odds for menthol cigarette use among those smoking cigarettes, stratified by that sociodemographic characteristic to explore the nature of the interaction in more detail. Since we modeled age continuously using restricted cubic splines, we graphically presented the adjusted prevalence of smoking menthol or non-flavored cigarettes and the adjusted prevalence of menthol cigarette use across the age range.

#### Trends in Menthol Cigarette Smoking and Predominant RYO Use (RQs 3–4)

We used logistic regression with a nonlinear term for time (using restricted cubic splines, as described above) to estimate monthly time trends (from October 2020 to October 2023) in: (1) the prevalence of predominantly smoking RYO cigarettes among adults who smoke in Great Britain, stratified by whether someone smoked menthol or non-flavored cigarettes, and (2) the prevalence of smoking menthol cigarettes among those predominantly smoking RYO cigarettes. To put the latter trend into context, we also assessed the prevalence of smoking menthol cigarettes among (1) those predominantly smoking factory-made cigarettes (not preregistered), (2) all who smoke cigarettes, and (3) the total population. We plotted the modeled estimates and their 95% CIs and assessed trends descriptively (exact values are presented in Supplementary Material).

## Results

Between October 2020 and October 2023, 84 003 participants took part in the survey of whom 81 293 participants (96.8%) had available data on all relevant variables (unweighted, see Table S1 for missing values per variable). The median age was 49 years. Among the participants, 51.0% identified as female, 48.5% as male, 0.6% as non-binary, 13.1% belonged to ethnic minority groups, 43.9% were from less advantaged social grades (C2DE), 86.3% were from England, 8.8% from Scotland, 4.9% from Wales, and 13.4% currently smoked cigarettes. Absolute numbers and unweighted data are presented in Table S2. Most people who were categorized as either smoking predominantly RYO or factory-made cigarettes smoked this type of cigarette exclusively (among those predominantly smoking RYO, 95.5% exclusively used RYO, and among those predominantly smoking factory-made, 94.7% exclusively used factory-made cigarettes).

### Associations Overall and by Subgroups

**Figure 1.**
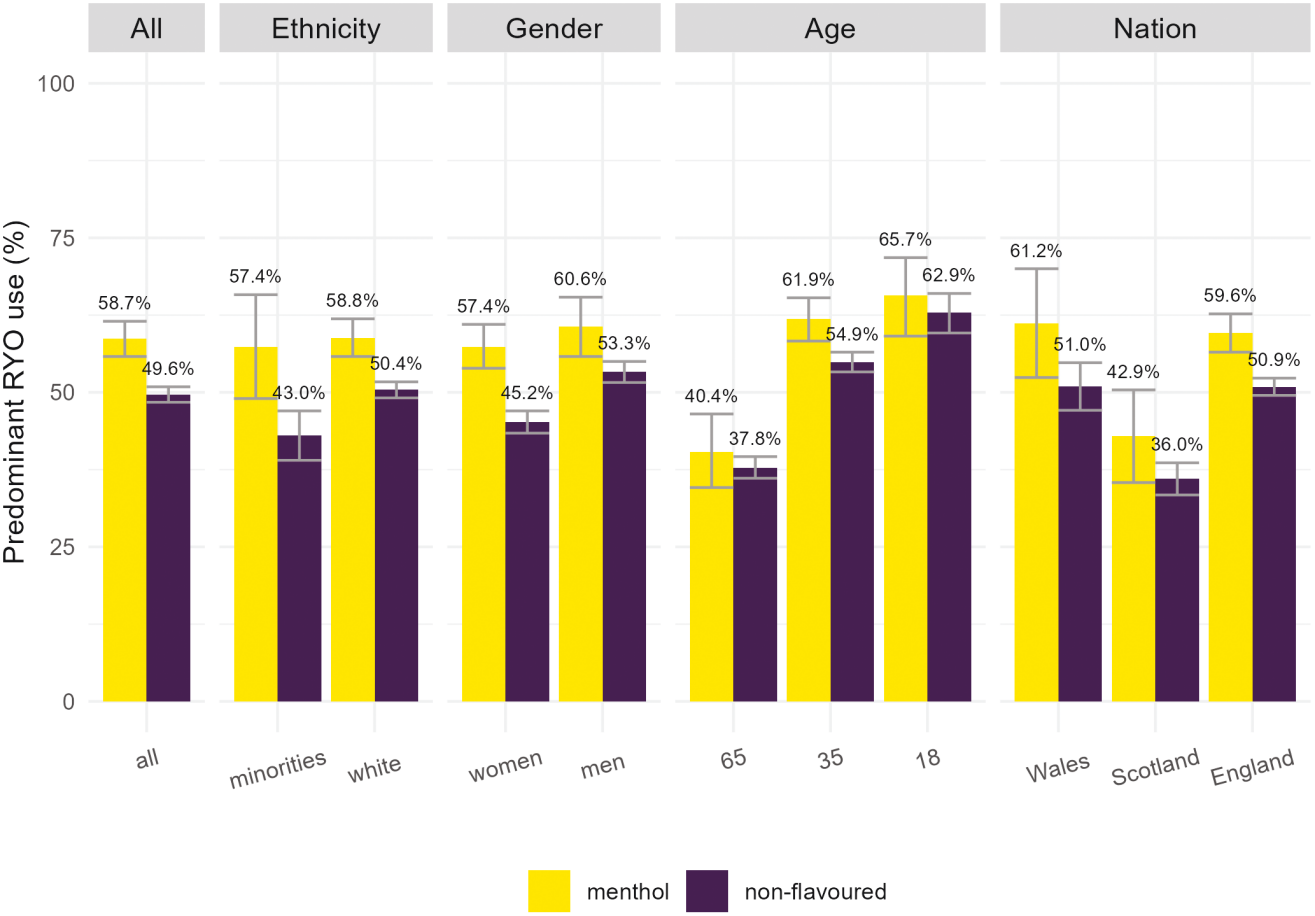
Percentage predominantly using roll-your-own tobacco among people smoking menthol or non-flavored cigarettes, stratified by sociodemographic characteristics (*n*_unweighted_ = 9790).

Overall, 58.7% (95% CI: 55.8 to 61.5) of people who smoked menthol cigarettes and 49.6% (95% CI: 48.4 to 50.9) of people who smoked non-flavored cigarettes used predominantly RYO tobacco (Figure 1 and Table S3, unweighted Figure S1). For most subgroups, the percentage of people using predominantly RYO was higher when smoking menthol than non-flavored cigarettes. People who smoked menthol cigarettes had 1.30 (95% CI: 1.14 to 1.49) times higher odds of predominantly using RYO cigarettes than people who smoked non-flavored cigarettes when adjusting for covariates (Table 1, unweighted data in Table S4).

**Table 1.** Associations Between Predominant RYO Use and Menthol Cigarette Smoking and Interactions With Sociodemographic Characteristics (*n*_unweighted_ = 9790)

	Predominant RYO use	
	OR (95% CI)	OR_adj_^*^ (95% CI)
Menthol cigarette smoking overall	1.44 (1.27 to 1.64)	1.30 (1.14 to 1.49)
Interaction with ethnicity: minorities (ref.: White)	1.27 (0.85 to 1.91)	1.56 (1.03 to 2.36)
Interaction with gender: women (ref.: men)	1.21 (0.93 to 1.58)	1.26 (0.95 to 1.65)
Interaction with age^1^: 18 (ref.: 65)	1.02 (0.83 to 1.20)	0.98 (0.79 to 1.17)
Interaction with age^1^: 35 (ref.: 65)	1.20 (1.01 to 1.38)	1.18 (1.01 to 1.35)
Interaction with nation: Scotland (ref.: England)	0.94 (0.66 to 1.34)	0.97 (0.67 to 1.41)
Interaction with nation: Wales (ref.: England)	1.07 (0.70 to 1.63)	1.03 (0.66 to 1.62)

^*^Adjusted for age, gender, ethnicity, nation, and social grade.

^1^Estimates for age are derived from restricted cubic splines modeling. Abbreviations: CI = confidence interval; OR = odds ratio; OR_adj_ = adjusted odds ratio; ref = reference; RYO = roll-your-own.

This association depended on age and ethnicity, with middle-aged people and ethnic minorities having higher odds of predominantly using RYO when smoking menthol cigarettes than older and White people (35 years vs. 65 years OR_adj_ = 1.18, 95% CI: 1.01to 1.35; ethnic minorities vs. White OR_adj_ = 1.56, 95% CI: 1.03 to 2.36). Middle-aged adults (~30 + years) who smoked menthol cigarettes had a higher prevalence of predominant RYO use than those who smoked non-flavored cigarettes, while young adults (~18–29 years) and those aged around 60 and older both had similar prevalence estimates when adjusting for covariates (Figure S2). Overall, there was a substantial age gradient for menthol cigarette use among those who smoke cigarettes, with the youngest adults having a roughly 10 times higher prevalence than the oldest (22% vs. 2%) when adjusting for covariates (Figure S3).

People from ethnic minority groups who smoked menthol cigarettes had 1.79 (95% CI: 1.21to 2.64) times higher odds of predominantly using RYO than those who smoked non-flavored cigarettes when adjusting for covariates, while for White people, the adjusted odds ratio was 1.24 (95% CI: 1.08 to 1.43). A more granular analysis by ethnicity showed Asian or mixed White/Asian people had 2.10 (95% CI: 1.00 to 4.43) times higher odds and Black or mixed White/Black people had 1.50 (95%: 0.76, 2.94) times higher odds than White people (Table S5). However, due to the small sample sizes within the ethnic minority groups, these estimates are somewhat uncertain. In general, among those smoking cigarettes, people from ethnic minority groups had similar odds of using menthol cigarettes compared to White people (OR_adj_ = 0.93, 95% CI: 0.75, 1.14).

There was a tendency for women to have higher odds of predominant RYO use when smoking menthol cigarettes than men, but this association was uncertain (OR_adj_ = 1.26, 95% CI: 0.95 to 1.65). The odds of predominant RYO use for people smoking menthol cigarettes compared to those smoking non-flavored cigarettes did not differ by nation. However, RYO use appeared to be generally less common in Scotland compared to England and Wales (Figure 1).

### Trends in Menthol Cigarette Smoking and Predominant RYO Use

**Figure 2.**
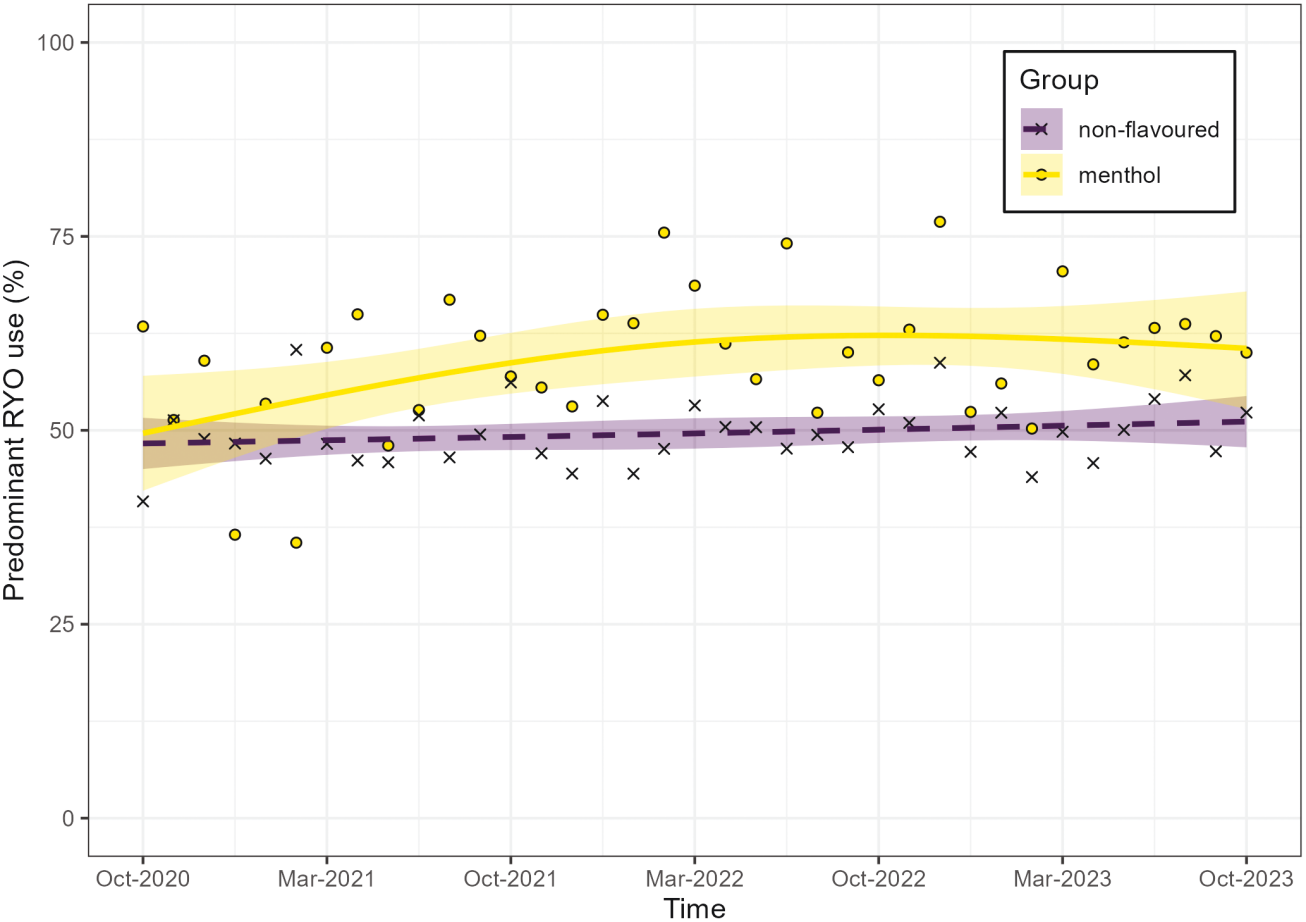
Modeled trends in predominant roll-your-own use among those smoking menthol cigarettes (*n*_unweighted_ = 1407) compared to those smoking non-flavored cigarettes (*n*_unweighted_ = 8413) between October 2020 and October 2023. Shaded areas indicate 95% CIs and points show unmodelled estimates.

While the percentage of people predominantly using RYO cigarettes was comparable between those smoking menthol and those smoking non-flavored cigarettes in October 2020, predominant RYO use became more common among those smoking menthol cigarettes over the subsequent three years (Figure 2, unweighted Figure S4). Between October 2020 and October 2023, the percentage of people reporting menthol cigarette smoking who were predominantly using RYO cigarettes increased until mid-2022, from 49.6% (95% CI: 42.2 to 57.0) in October 2020 to 61.9% (95% CI: 57.5 to 66.0) in June 2022 (Table S6, unweighted data in Table S7). In the time between June 2022 and October 2023, it remained relatively stable at around 60%. By contrast, among people smoking non-flavored cigarettes, the percentage predominantly using RYO cigarettes did not change significantly between October 2020 and October 2023, from 48.3% (95% CI: 45.0 to 51.6) to 51.1% (95% CI: 47.8 to 54.4, see Table S6, unweighted data in Table S7).

**Figure 3.**
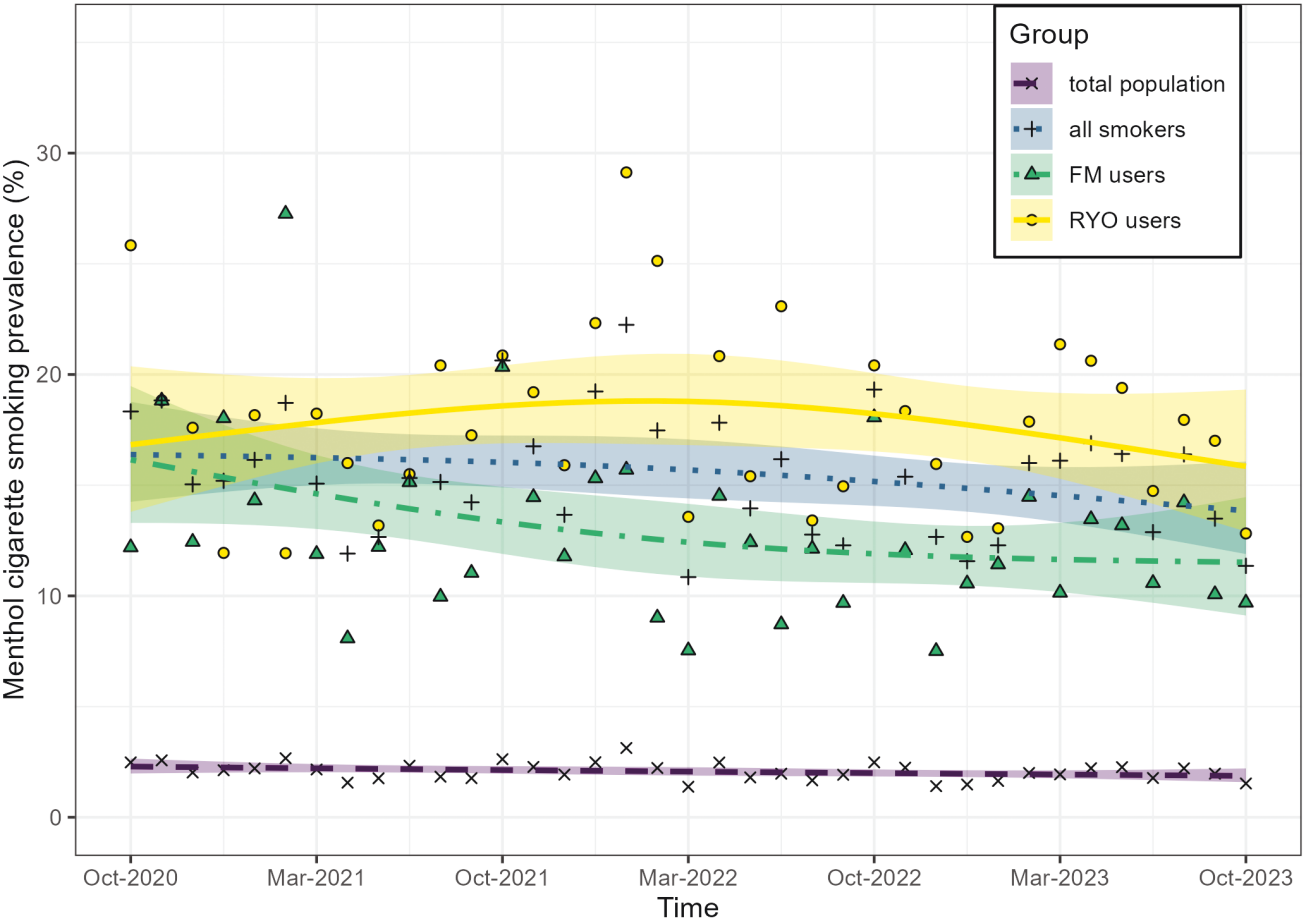
Menthol cigarette smoking prevalence among the adult population (*N*_unweighted_ = 82120), all who smoke cigarettes (*n*_unweighted_ = 10710), and those predominantly smoking roll-your-own cigarettes (*n*_unweighted_ = 5401) between October 2020 and October 2023. Shaded areas indicate 95% CIs and dots show unmodelled estimates.

There was a minimal decline in the percentage of people reporting smoking menthol cigarettes in the total population from 2.3% (95% CI: 2.0 to 2.7) in October 2020 to 1.9% (95% CI: 1.6 to 2.2) in October 2023 (see Table S8, unweighted data in Table S9; Figure 3, unweighted Figure S5). This minimal decline in menthol cigarette smoking prevalence was also seen among all people who smoked, from 16.4% (95% CI: 14.2 to 18.8) in October 2020 to 13.8% (95% CI: 11.9 to 16.1) in October 2023 (see Table S8, unweighted data in Table S9). However, this overall small change among all who smoked cigarettes masked divergent trends in menthol cigarette smoking prevalence among people who smoked predominantly RYO versus factory-made cigarettes (Figure 3). For predominant RYO use, there was a slight increase until 2022, followed by a slight decline, while among predominant factory-made cigarette users, the prevalence appeared to decline slightly until 2022 and then plateaued.

## Discussion

### Summary of Findings

This study extends previous findings^8^ by showing that up until October 2023, a substantial proportion of smoking adults in Great Britain reported using menthol cigarettes. In Great Britain, between October 2020 and October 2023, people who smoked menthol cigarettes had 30% higher odds of predominant RYO use compared with people who smoked non-flavored cigarettes. Across all demographic subgroups, people who use menthol cigarettes were more likely to predominantly use RYO. However, there were certain groups where RYO use was especially prominent in menthol smokers. These included adults who were middle-aged and from ethnic minorities. The strengthening in the association between predominant RYO use and menthol cigarette smoking, and the fact that over half of people smoking menthol cigarettes predominantly use RYO tobacco now, suggest that the availability of menthol accessories for RYO tobacco may have undermined objectives of the 2020 menthol ban. During the first half of the study period (from October 2020 until roughly June 2022), among people smoking menthol cigarettes, the proportion who predominantly used RYO cigarettes increased and then stayed at a higher level. At the same time, for people who smoked non-flavored cigarettes, the proportion who used predominantly RYO cigarettes remained relatively constant throughout. A few months after the menthol ban came into force, about the same proportions of people smoked menthol factory-made cigarettes and menthol RYO cigarettes, but over time, a higher proportion smoked the latter. These results show that RYO tobacco has become especially popular among people who smoke menthol cigarettes, with the majority (~60%) now reporting predominantly using RYO.

### Comparison to Existing Literature and Implications

Kyriakos et al.^11^ found that, in 2021, among 16-to-19-year-olds in England, the most common option for menthol cigarette smoking was menthol accessories in combination with RYO tobacco. Our results showed that, in general, young people are more likely to use RYO tobacco than factory-made cigarettes irrespective of whether they smoke menthol or non-flavored cigarettes. Further, our study indicates that the use of RYO tobacco and menthol accessories might be particularly popular among middle-aged adults. It is possible that older people who had been using factory-made menthol cigarettes for a long time responded differently to the ban than middle-aged adults. Namely, older people may have been more likely to seek factory-made menthol replacement cigarettes that are similar to the cigarettes they previously smoked (eg, the products under the “New Dual” range from Japan Tobacco International that were introduced as replacement products for their menthol cigarettes following the ban^14^), while among middle-aged adults a larger proportion switched to RYO tobacco. Both studies, ours and the one by Kyriakos et al.,^11^ are consistent in suggesting that there might be a higher menthol accessory use among ethnic minority groups. A potential explanation for these results is that advertising for menthol accessories may be more targeted towards ethnic minorities. However, our study did not show that ethnic minorities who smoke are generally more likely to use menthol cigarettes than White people.

It is also important to note that only factory-made cigarettes and loose tobacco for RYO cigarettes have been included in the menthol ban.^4^ The explanations for exempting other tobacco products, such as cigarillos, were low sales volumes or consumption among young people.^5^ However, new menthol cigarillos have since come onto the market.^34^ These are very similar to the factory-made menthol cigarettes, which are no longer available due to the ban in the UK.^34^ Recent research found that, since 2020 (roughly at the time when the menthol ban was implemented, but also the start of the COVID pandemic), there has been an increase in non-cigarette tobacco smoking in England.^35^ There is therefore reason to suspect that, in addition to menthol cigarettes (such as those examined in this study), menthol cigarillos are also used by some people who prefer to smoke mentholated tobacco over non-flavored tobacco.

We also found that in Scotland fewer people predominantly used RYO tobacco, for both menthol and non-flavored cigarette smoking, compared to people in England and Wales, which is in line with another study on RYO use using data from 2001 to 2008.^33^ The finding that menthol cigarette smoking is less prevalent in Scotland than in England may, therefore, be explained by the fact that fewer people generally use RYO tobacco (and thus menthol accessories) than in the other nations, rather than by the more comprehensive display ban as we previously hypothesized.^8^

Future studies could further explore how menthol accessories are advertised in Great Britain and to which target groups. As this study provides evidence that current menthol cigarette smoking in Great Britain might be driven by RYO tobacco and menthol accessories, particularly among priority groups (such as ethnic minorities), policymakers should consider including any tobacco-related products (eg, filters, rolling paper, crush balls) in the menthol ban, or at least restrict advertising of related products to reduce inequalities. As menthol cigarette smoking prevalence appears to have been relatively stable since October 2020, new measures will be required to achieve further reductions in menthol cigarette smoking prevalence. Future research could also investigate whether menthol-flavored e-cigarettes could be an alternative for people currently smoking combustible mentholated cigarettes as a harm reduction measure.

### Limitations

The study has several limitations. It used a repeat cross-sectional design so cannot offer insight into changes in product use within individuals over time. It relied on self-reported data which could bias the estimated absolute prevalence but relative changes over time and within group differences should be unaffected by it. Further, our study did not collect data on menthol cigarette smoking from before or at the time the ban was introduced. The question about the flavor of the cigarettes people usually smoke was added to the survey in July 2020. The Smoking Toolkit Study initially collected data from England and expanded to Scotland and Wales in October 2020. We decided to include all data for Great Britain from October 2020 onwards in the study (the prevalence of menthol use among adults who smoked cigarettes in England surveyed between July to September 2020 was 17.7%, similar to the prevalence we observed across Great Britain in October 2020). Therefore, we cannot draw any comparisons between the period after full implementation and the period before or at the time of the ban.

Another limitation is that the survey does not include any questions about the specific type of menthol cigarette people were smoking. Therefore, we could only indirectly infer that people who reported using predominantly RYO tobacco and smoking cigarettes with menthol flavor would likely be using RYO cigarettes with menthol accessories. Qualitative research would be useful to gain more insight into the specific types of products being used by people who smoke menthol cigarettes (including those who predominantly use RYO tobacco). In addition, we were unable to assess the prevalence of menthol cigarillo consumption because the survey did not include specific questions on cigarillo use and menthol cigarillos in particular. However, it is important to examine the use of these products since many came onto the market in response to the menthol ban.^34^ Furthermore, the study might not be sufficiently powered to identify differences in some of the subgroup analyses. This also limited our ability to assess differences by ethnicity. We only distinguished between White people and ethnic minorities in the main analysis. A more granular analysis is presented in Supplementary Material, but it is based on small sample sizes for different ethnic groups so results should be interpreted with some caution. Additionally, as data were drawn from a telephone household survey, only participants living in private households and able to take a phone call were included in the study.

## Conclusions

In Great Britain, people who smoke menthol cigarettes are more likely to report that they predominantly use RYO tobacco than people who smoke non-flavored cigarettes. There has been an increase in predominant RYO use among people smoking menthol cigarettes from approximately 50% at the end of 2020 to approximately 60% since mid-2022. As overall menthol smoking hardly changed since October 2020 (and increased among RYO tobacco users), it appears that the intended impact of the ban (ie, reducing menthol smoking) has been in part undermined by more people using RYO with menthol accessories.

## Supplementary material

Supplementary material is available at *Nicotine and Tobacco Research* online.

ntae217_suppl_Supplementary_Materials

## Data Availability

Data and command syntax for the statistical analyses will be available upon publication on the Open Science Framework (https://osf.io/y8sge/).
