# Supplementary material for: Roll-Your-Own Tobacco Use Among People Smoking Menthol Cigarettes in Great Britain, 2020-2023: A Population-Based Survey
Source: Nicotine Tob Res. 2024 Sep 10;27(7):1200–8. doi: 10.1093/ntr/ntae217 (PMC12187076; doi:10.1093/ntr/ntae217)
Supplement: ntae217_suppl_Supplementary_Materials [file ntae217_suppl_supplementary_materials.pdf]

SUPPLEMENTARY MATERIAL

Supplementary to article: "Roll-your-own tobacco use among people smoking menthol cigarettes in Great Britain, 2020-2023: a population-based survey "

1. Background about the menthol ban

The 2014 Tobacco Products Directive of the European Union included a ban on menthol cigarettes, in line with the World Health Organization's Framework Convention on Tobacco Control.[1,2] The Directive prohibits tobacco products with characterising flavours, such as menthol, that notably alter the smell or taste of the product.[2] The exact definition of characterising flavours in this context is: "a clearly noticeable smell or taste other than one of tobacco, resulting from an additive or a combination of additives, including, but not limited to, fruit, spice, herbs, alcohol, candy, menthol or vanilla, which is noticeable before or during the consumption of the tobacco product".[1] The Directive also applies to the United Kingdom (UK), which incorporated this ban into British law after the country left the European Union.[3] Generally, there was a 2-year implementation period from 2014 to 2016. However, the Directive allowed for an additional 4-year grace period for "tobacco products with a characterising flavour whose Union-wide sales volumes represent 3% or more in a particular product category", which applied to menthol cigarettes.[1] Therefore, the menthol ban became effective in May 2020 in the European Union and the UK.[2]

Under the Directive, adding flavours such as menthol to tobacco is not fundamentally prohibited. Such additives are allowed "as long as they do not result in a characterising flavour or increase the addictiveness, toxicity or CMR [carcinogenic, mutagenic or reprotoxic] properties of the product."[1] Two problems with this specification are that it is difficult to determine whether a cigarette possesses characterising flavours and menthol has sensory properties that can be perceived even at low concentrations.[4,5]

Additionally, the Directive stated that the prohibition of characterising flavours only applies to factory-made (FM) cigarettes and roll-your-own (RYO) tobacco.[1] Other products were exempted with the explanation that the Directive focussed on young people who primarily consumed these two products.[1] Due to this exemption, new products with menthol flavour could enter the market, including mentholated cigarillos and menthol-flavoured tobacco accessories.[6,7] Such products are also not included in other tobacco control policies in the UK. For example, standardised packaging requirements, including minimum pack sizes, do not apply to cigarillos.[7] Tobacco accessories, including rolling paper and filters, are not subject to the same strict regulations on marketing and promotion as tobacco products.[8] For example, tobacco accessories can be openly on display in shops and advertisements are allowed as long as they do not encourage uptake or increased consumption of cigarettes.[8] In Scotland, the point-of-sale display ban varies slightly from England and Wales by including all tobacco-related products in the ban.[9,10]

2. Missing values

Table S1: Proportion of data missing for each variable ($N_{unweighted}$=84,003).

Variable	Missing values, n (%)
Age	0 (0)
Gender	182 (0.2)
Ethnicity	657 (0.8)
Nation	0 (0)
Social grade	0 (0)
Cigarette smoking	631 (0.8)
Flavoured cigarettes (cigarette smoking n = 11,299)*	235 (2.1)
Predominantly RYO (cigarette smoking n = 11,299)	979 (8.7)

* n=240 participants stated that they smoked tobacco and some other flavour. These were additionally removed from the analysis. Abbreviation: RYO, roll-your-own.

3. Unweighted and weighted participant characteristics

Table S2: Characteristics of survey participants between October 2020 and October 2023, unweighted vs. weighted ($N_{unweighted}$=81,293).

Characteristic	Unweighted	Weighted
Age, median (IQR)	53 (36, 66)	49 (33, 64)
Female, n (%)	41359 (50.9)	41287 (51.0)
Male, n (%)	39880 (48.5)	39249 (48.5)
Non-binary, n (%)	467 (0.6)	467 (0.6)
Ethnic minorities, n (%)	9211 (11.3)	10608 (13.1)
Social grades C2DE, n (%)	27733 (34.1)	35557 (43.9)
England, n (%)	57618 (70.9)	69906 (86.3)
Scotland, n (%)	15301 (18.8)	7100 (8.8)
Wales, n (%)	8374 (10.3)	3997 (4.9)
Smoking cigarettes, n (%)	9883 (12.2)	10857 (13.4)

Abbreviation: IQR, interquartile range.

4. Associations between predominant roll-your-own (RYO) use and menthol cigarette smoking

Table S3: Percentage predominantly using RYO tobacco among people smoking menthol or non-flavoured cigarettes and stratified by sociodemographic characteristics ($n_{unweighted}$=9790).

Sociodemographic characteristic	Subgroup	Predominant RYO use	
		Menthol, % (95% CI)	Non-flavoured (95% CI)
All	–	58.7 (55.8-61.5)	49.6 (48.4-50.9)
Ethnicity	White	58.8 (55.8-61.9)	50.4 (49.1-51.7)
	Minorities	57.4 (49.0-65.9)	43.0 (39.0-47.0)
Gender	Men	60.6 (55.8-65.4)	53.9 (51.6-55.0)
	Women	57.4 (53.9-61.0)	45.2 (43.4-47.0)
Age	18 years	65.7 (59.1-71.8)	62.9 (59.6-66.1)
	35 years	61.9 (58.4-65.3)	54.9 (53.3-56.5)
	65 years	40.4 (34.6-45.5)	37.8 (36.1-39.6)
Nation	England	59.6 (56.5-62.7)	50.9 (49.5-52.3)
	Scotland	42.9 (35.4-50.4)	36.0 (33.4-38.6)
	Wales	61.2 (52.4-70.0)	51.0 (47.1-54.8)

Abbreviations: CI, confidence interval; RYO, roll-your-own.

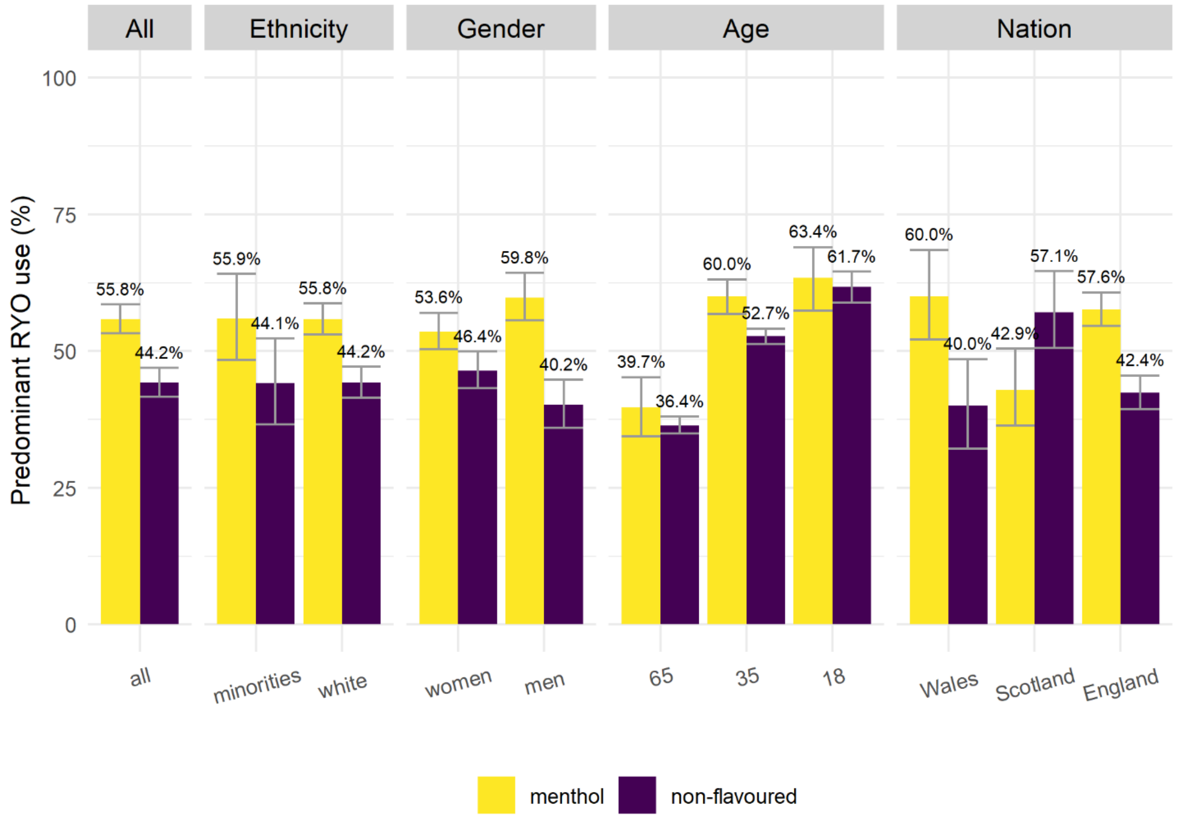

Figure S1: Unweighted percentage predominantly using RYO tobacco among people smoking menthol or non-flavoured cigarettes, stratified by sociodemographic characteristics ($n_{unweighted}$=9790).

Table S4: Unweighted associations between predominant RYO use and menthol cigarette smoking and interactions with sociodemographic characteristics ($n_{unweighted}$=9790).

	**Predominant RYO use**	
	OR (95% CI)	OR_{adj}* (95% CI)
Menthol smoking overall	1.46 (1.30-1.63)	1.33 (1.18-1.49)
Interaction with ethnicity: minorities (ref.: White)	1.17 (0.82-1.69)	1.43 (0.99, 2.07)
Interaction with gender: women (ref.: men)	1.15 (0.91-1.46)	1.20 (0.95-1.53)
Interaction with age: 18 (ref.: 65)	0.94 (0.77-1.10)	0.90 (0.74-1.07)
Interaction with age: 35 (ref.: 65)	1.18 (1.03-1.32)	1.18 (1.04-1.32)
Interaction with nation: Scotland (ref.: England)	1.00 (0.72-1.38)	1.04 (0.74-1.46)
Interaction with nation: Wales (ref.: England)	1.09 (0.74-1.61)	1.08 (0.73-1.61)

*Adjusted for age, gender, ethnicity, nation, and social grade. Abbreviations: CI, confidence interval; OR, odds ratio, OR_{adj}, adjusted odds ratio; ref, reference group; RYO, roll-your-own.

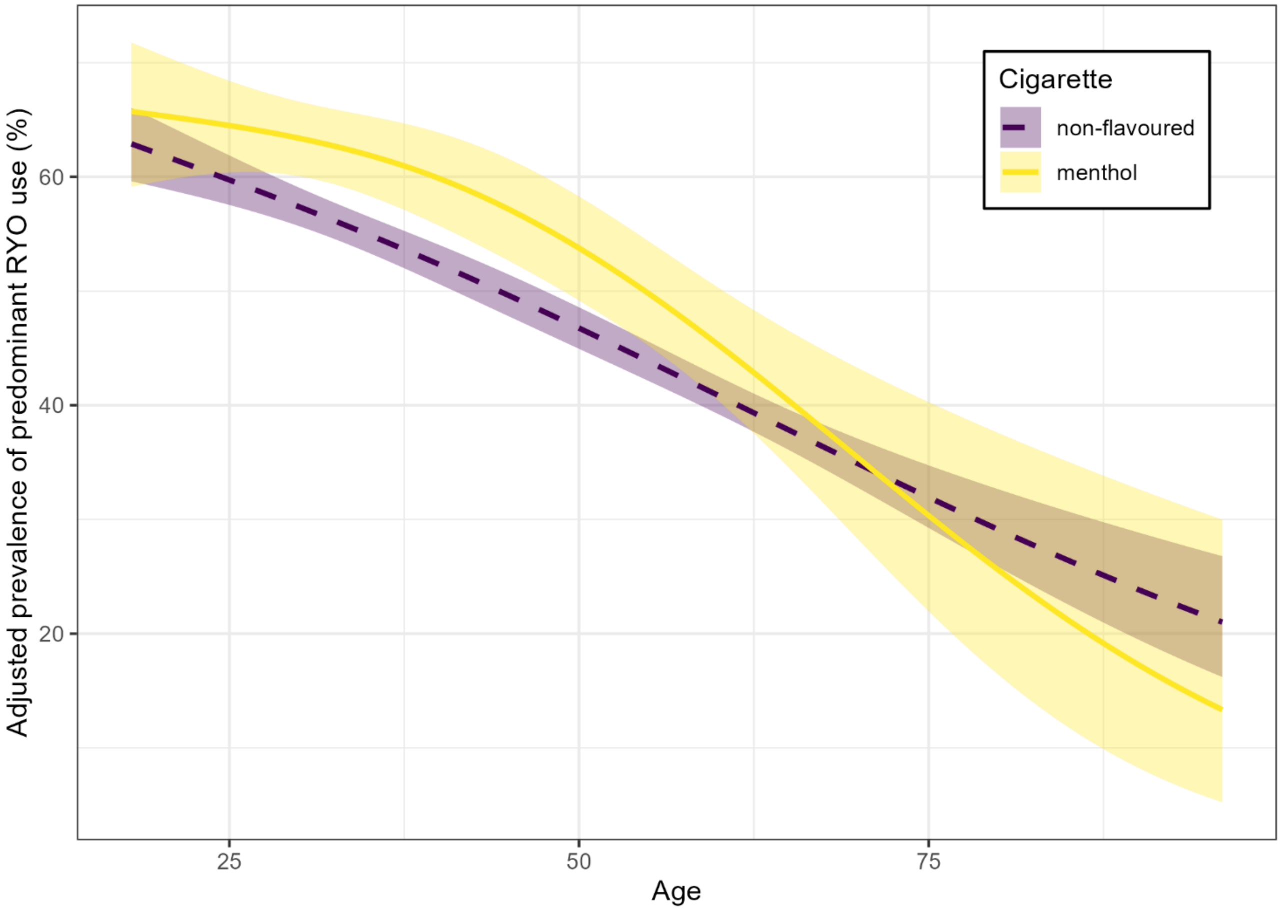

Figure S2: Modelled weighted and adjusted prevalence of predominant RYO use across the age range, stratified by type of cigarette smoked ($n_{unweighted}$=9790). Shaded areas indicate 95% confidence intervals.

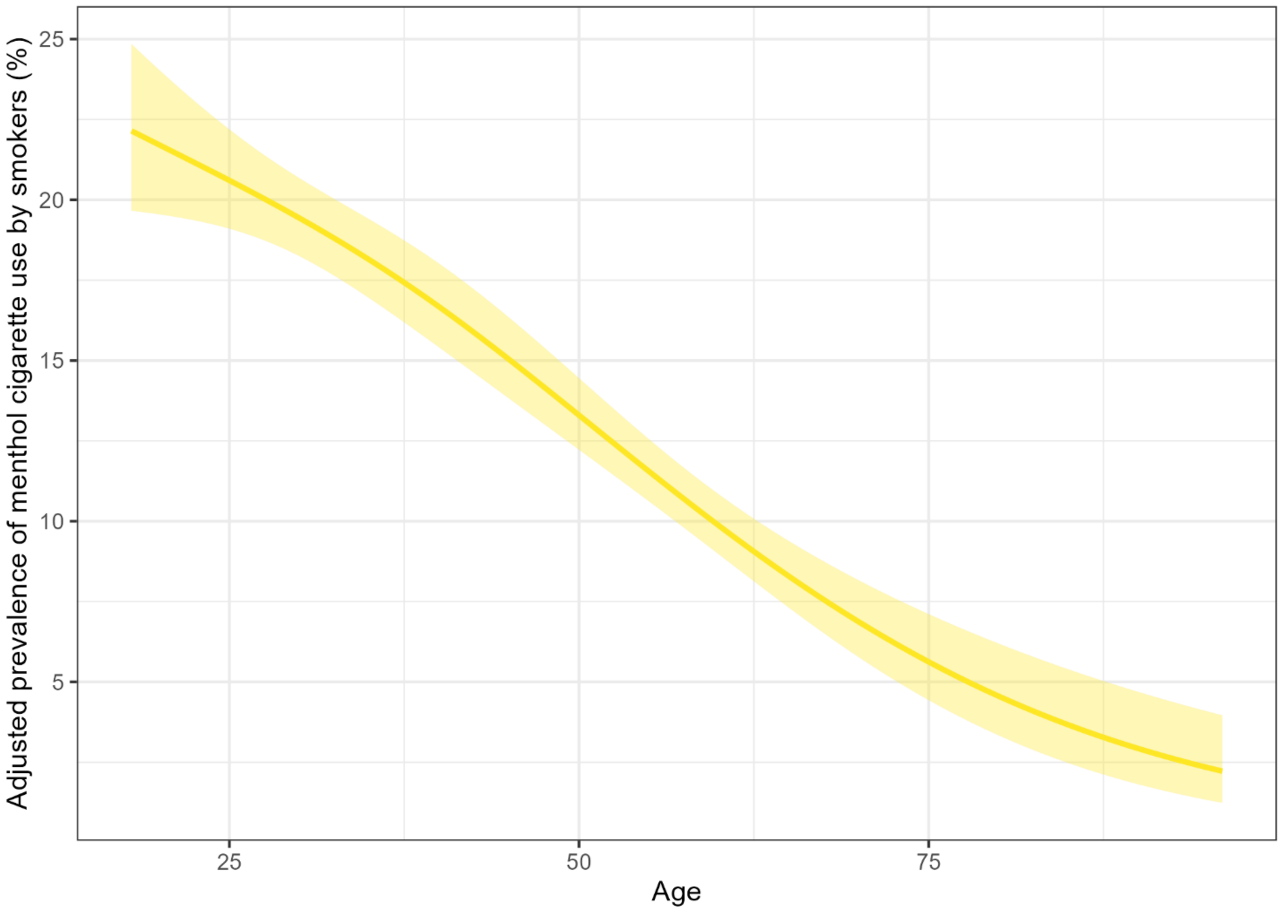

Figure S3: Modelled weighted and adjusted prevalence of predominant menthol cigarette use among people who smoke cigarettes across the age range ($n_{unweighted}$=9790). Shaded area indicates 95% confidence intervals.

Table S5: Associations between predominant RYO use and interactions between menthol cigarette smoking and ethnicity ($n_{unweighted}$=9790, reference group for ethnicity: White $n_{unweighted}$=8842).

		**Predominant RYO use**		
	$n_{unweighted}$	% (95% CI)	OR (95% CI)	OR_{adj}* (95% CI)
White	864	58.8 (55.8-61.8)	Reference	Reference
Asian or mixed White/Asian	385	55.2 (38.5-72.0)	1.65 (0.79-3.45)	2.10 (1.00-4.43)
Black or mixed White/Black	256	46.8 (47.7-74.4)	1.27 (0.65-2.46)	1.50 (0.76-2.94)
Other	307	55.4 (41.3-69.4)	0.82 (0.43-1.57)	0.98 (0.50, 1.92)

*Adjusted for age, gender, nation, and social grade. Abbreviations: CI, confidence interval; OR, odds ratio, OR_{adj}, adjusted odds ratio; RYO, roll-your-own.

5. Predominant RYO use among those smoking menthol vs. non-flavoured cigarettes

Table S6: Weighted modelled estimates in predominant RYO use among those smoking menthol cigarettes ($n_{unweighted}$=1470) compared to those smoking non-flavoured cigarettes ($n_{unweighted}$=8413) between October 2020 and October 2023.

Month-year	Predominant RYO use, % (95% CI)	
	Menthol cigarette smoking	Non-flavoured cigarette smoking
Oct-20	49.6 (42.2-57.0)	48.3 (45.0-51.6)
Nov-20	50.5 (43.7-57.2)	48.4 (45.4-51.4)
Dec-20	51.3 (45.1-57.5)	48.4 (45.7-51.2)
Jan-21	52.1 (46.5-57.7)	48.5 (46.0-51.0)
Feb-21	53.0 (47.8-58.0)	48.6 (46.3-50.8)
Mar-21	53.8 (49.0-58.4)	48.6 (46.6-50.7)
Apr-21	54.5 (50.2-58.8)	48.7 (46.8-50.6)
May-21	55.3 (51.2-59.3)	48.8 (47.0-50.5)
Jun-21	56.1 (52.2-59.9)	48.9 (47.2-50.5)
Jul-21	56.8 (53.0-60.5)	48.9 (47.3-50.6)
Aug-21	57.4 (53.6-61.2)	49.0 (47.4-50.6)
Sep-21	58.1 (54.2-61.9)	49.1 (47.4-50.7)
Oct-21	58.7 (54.7-62.6)	49.1 (47.5-50.8)
Nov-21	59.3 (55.2-63.2)	49.2 (47.5-51.0)
Dec-21	59.8 (55.6-63.9)	49.3 (47.5-51.1)
Jan-22	60.3 (55.9-64.4)	49.4 (47.5-51.2)
Feb-22	60.7 (56.3-64.9)	49.4 (47.5-51.4)
Mar-22	61.1 (56.6-65.3)	49.5 (47.6-51.5)
Apr-22	61.4 (56.9-65.7)	49.6 (47.6-51.6)
May-22	61.7 (57.2-65.9)	49.7 (47.7-51.6)
Jun-22	61.9 (57.5-66.0)	49.8 (47.8-51.7)
Jul-22	62.0 (57.8-66.1)	49.8 (48.0-51.7)
Aug-22	62.1 (58.0-66.1)	49.9 (48.1-51.7)
Sep-22	62.2 (58.2-66.0)	50.0 (48.2-51.8)
Oct-22	62.2 (58.4-66.0)	50.1 (48.4-51.8)
Nov-22	62.2 (58.4-65.9)	50.2 (48.5-51.8)
Dec-22	62.2 (58.4-65.8)	50.3 (48.6-51.9)
Jan-23	62.1 (58.3-65.8)	50.3 (48.7-52.0)
Feb-23	62.0 (58.1-65.8)	50.4 (48.7-52.1)
Mar-23	61.9 (57.7-65.9)	50.5 (48.7-52.3)

Month-year	Predominant RYO use, % (95% CI)	
	Menthol cigarette smoking	Non-flavoured cigarette smoking
Apr-23	61.7 (57.3-66.0)	50.6 (48.7-52.5)
May-23	61.6 (56.7-66.2)	50.7 (48.6-52.8)
Jun-23	61.4 (56.0-66.5)	50.8 (48.5-53.0)
Jul-23	61.2 (55.3-66.8)	50.9 (48.4-53.4)
Aug-23	61.0 (54.5-67.1)	50.9 (48.2-53.7)
Sep-23	60.8 (53.6-67.5)	51.0 (48.0-54.1)
Oct-23	60.6 (52.7-67.9)	51.1 (47.8-54.4)

Abbreviations: CI, confidence interval; RYO, roll-your-own.

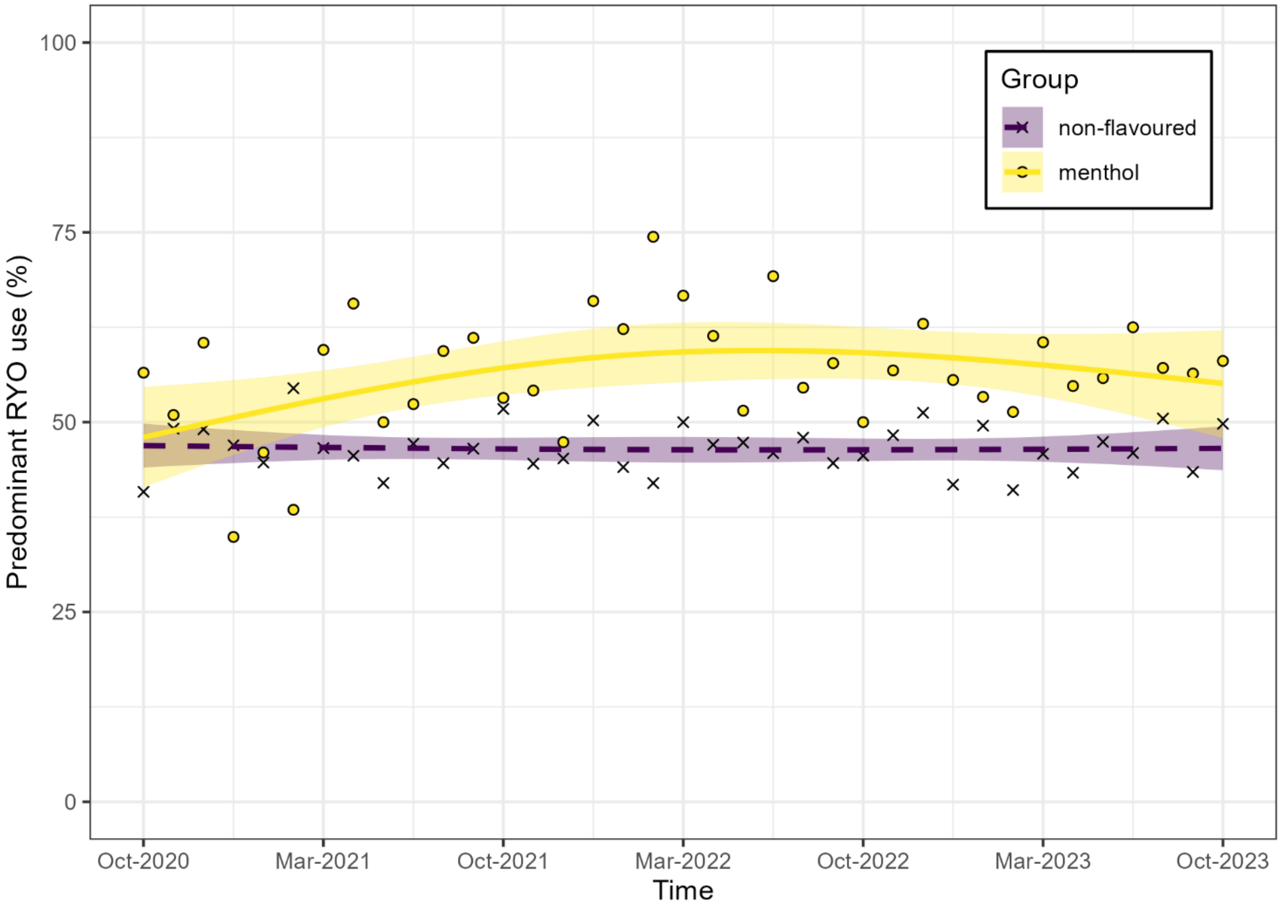

Figure S4: Unweighted modelled trends in predominant RYO use among those smoking menthol cigarettes ($n_{unweighted}$=1470) compared to those smoking non-flavoured cigarettes ($n_{unweighted}$= 8413) between October 2020 and October 2023. Shaded areas indicate 95% confidence intervals and dots show unmodelled estimates. Abbreviation: RYO, roll-your-own.

Table S7: Unweighted, modelled estimates in predominant RYO use among those smoking menthol cigarettes ($n_{unweighted}$=1470) compared to those smoking non-flavoured cigarettes ($n_{unweighted}$=8413) between October 2020 and October 2023.

**Month-year**	**Predominant RYO use, % (95% CI)**	
	Menthol cigarette smoking	Non-flavoured cigarette smoking
Oct-20	48.0 (41.5-54.6)	46.9 (44.0-49.8)
Nov-20	48.9 (42.9-54.9)	46.9 (44.2-49.5)
Dec-20	49.8 (44.3-55.2)	46.8 (44.4-49.2)
Jan-21	50.6 (45.7-55.5)	46.8 (44.6-49.0)
Feb-21	51.5 (47.0-55.9)	46.8 (44.8-48.7)
Mar-21	52.3 (48.2-56.3)	46.7 (44.9-48.5)
Apr-21	53.1 (49.3-56.8)	46.7 (45.0-48.3)
May-21	53.9 (50.3-57.4)	46.6 (45.1-48.2)
Jun-21	54.6 (51.2-58.0)	46.6 (45.2-48.1)
Jul-21	55.3 (52.0-58.6)	46.6 (45.2-48.0)
Aug-21	56.0 (52.6-59.3)	46.5 (45.1-47.9)
Sep-21	56.6 (53.1-60.0)	46.5 (45.1-47.9)
Oct-21	57.2 (53.6-60.6)	46.5 (45.0-47.9)
Nov-21	57.7 (54.0-61.3)	46.5 (44.9-48.0)
Dec-21	58.1 (54.3-61.8)	46.4 (44.9-48.0)
Jan-22	58.5 (54.6-62.3)	46.4 (44.8-48.0)
Feb-22	58.8 (54.8-62.7)	46.4 (44.7-48.0)
Mar-22	59.1 (55.1-62.9)	46.4 (44.7-48.1)
Apr-22	59.2 (55.3-63.1)	46.4 (44.7-48.1)
May-22	59.4 (55.4-63.2)	46.4 (44.7-48.0)
Jun-22	59.4 (55.6-63.2)	46.4 (44.7-48.0)
Jul-22	59.4 (55.7-63.1)	46.4 (44.7-48.0)
Aug-22	59.4 (55.7-62.9)	46.4 (44.8-47.9)
Sep-22	59.3 (55.7-62.7)	46.4 (44.8-47.9)
Oct-22	59.1 (55.7-62.5)	46.4 (44.9-47.8)
Nov-22	59.0 (55.6-62.3)	46.4 (44.9-47.8)
Dec-22	58.7 (55.3-62.1)	46.4 (45.0-47.8)
Jan-23	58.5 (55.0-61.9)	46.4 (45.0-47.8)
Feb-23	58.2 (54.6-61.7)	46.4 (44.9-47.9)
Mar-23	57.9 (54.0-61.6)	46.4 (44.9-48.0)
Apr-23	57.5 (53.3-61.6)	46.4 (44.8-48.1)

Month-year	Predominant RYO use, % (95% CI)	
	Menthol cigarette smoking	Non-flavoured cigarette smoking
May-23	57.2 (52.6-61.6)	46.5 (44.7-48.3)
Jun-23	56.8 (51.8-61.6)	46.5 (44.5-48.5)
Jul-23	56.4 (50.9-61.7)	46.5 (44.3-48.7)
Aug-23	56.0 (49.9-61.8)	46.5 (44.1-48.9)
Sep-23	55.5 (48.9-62.0)	46.5 (43.9-49.2)
Oct-23	55.1 (47.9-62.1)	46.6 (43.7-49.4)

Abbreviations: CI, confidence interval; RYO, roll-your-own.

5. Menthol cigarette smoking among RYO users, smokers, total population

Table S8: Weighted modelled estimates in menthol cigarette smoking prevalence among the adult population ($N_{unweighted}$=81,293), all who smoke cigarettes ($n_{unweighted}$=9883), and those predominantly smoking RYO cigarettes ($n_{unweighted}$=4737) between October 2020 and October 2023.

Month-year	Menthol cigarette smoking, % (95% CI)			
	Total population	All smokers	FM users	RYO users
Oct-20	2.3 (2.0-2.7)	16.4 (14.2-18.8)	16.1 (13.3-19.5)	16.8 (13.8-20.4)
Nov-20	2.3 (2.0-2.6)	16.4 (14.4-18.5)	15.9 (13.3-18.9)	17.0 (14.2-20.2)
Dec-20	2.3 (2.0-2.6)	16.3 (14.6-18.3)	15.6 (13.3-18.3)	17.2 (14.6-20.1)
Jan-21	2.3 (2.0-2.5)	16.3 (14.7-18.1)	15.4 (13.2-17.7)	17.3 (15.0-20.0)
Feb-21	2.2 (2.0-2.5)	16.3 (14.8-17.9)	15.1 (13.2-17.2)	17.5 (15.3-19.9)
Mar-21	2.2 (2.0-2.4)	16.3 (14.9-17.7)	14.9 (13.1-16.8)	17.7 (15.7-19.9)
Apr-21	2.2 (2.0-2.4)	16.2 (15.0-17.6)	14.6 (13.0-16.4)	17.8 (16.0-19.8)
May-21	2.2 (2.0-2.4)	16.2 (15.1-17.4)	14.4 (12.9-16.0)	18.0 (16.3-19.8)
Jun-21	2.2 (2.0-2.4)	16.2 (15.1-17.4)	14.2 (12.7-15.7)	18.1 (16.5-19.9)
Jul-21	2.2 (2.0-2.3)	16.2 (15.1-17.3)	13.9 (12.5-15.5)	18.3 (16.6-20.0)
Aug-21	2.2 (2.0-2.3)	16.1 (15.0-17.3)	13.7 (12.3-15.2)	18.4 (16.8-20.1)
Sep-21	2.1 (2.0-2.3)	16.1 (15.0-17.2)	13.5 (12.1-15.1)	18.5 (16.8-20.2)
Oct-21	2.1 (2.0-2.3)	16.0 (14.9-17.2)	13.3 (11.9-14.9)	18.6 (16.9-20.4)
Nov-21	2.1 (2.0-2.3)	16.0 (14.8-17.2)	13.2 (11.7-14.8)	18.7 (16.9-20.6)
Dec-21	2.1 (1.9-2.3)	15.9 (14.7-17.2)	13.0 (11.5-14.7)	18.7 (16.9-20.7)
Jan-22	2.1 (1.9-2.3)	15.9 (14.6-17.2)	12.8 (11.3-14.5)	18.8 (16.9-20.8)
Feb-22	2.1 (1.9-2.3)	15.8 (14.6-17.2)	12.7 (11.1-14.4)	18.8 (16.9-20.9)
Mar-22	2.1 (1.9-2.3)	15.8 (14.5-17.1)	12.5 (11.0-14.3)	18.8 (16.8-20.9)
Apr-22	2.1 (1.9-2.3)	15.7 (14.4-17.1)	12.4 (10.9-14.2)	18.8 (16.8-20.9)
May-22	2.1 (1.9-2.2)	15.6 (14.3-17.0)	12.3 (10.8-14.0)	18.7 (16.8-20.9)

Month-year	Menthol cigarette smoking, % (95% CI)			
	Total population	All smokers	FM users	RYO users
Jun-22	2.0 (1.9-2.2)	15.5 (14.3-16.9)	12.2 (10.7-13.9)	18.7 (16.7-20.8)
Jul-22	2.0 (1.9-2.2)	15.4 (14.2-16.8)	12.1 (10.7-13.7)	18.6 (16.7-20.6)
Aug-22	2.0 (1.9-2.2)	15.4 (14.2-16.6)	12.0 (10.6-13.6)	18.5 (16.7-20.5)
Sep-22	2.0 (1.8-2.2)	15.3 (14.1-16.5)	12.0 (10.6-13.5)	18.4 (16.6-20.3)
Oct-22	2.0 (1.8-2.2)	15.2 (14.1-16.3)	11.9 (10.6-13.4)	18.2 (16.5-20.1)
Nov-22	2.0 (1.8-2.1)	15.1 (14.0-16.2)	11.8 (10.6-13.3)	18.1 (16.4-19.8)
Dec-22	2.0 (1.8-2.1)	15.0 (13.9-16.1)	11.8 (10.5-13.2)	17.9 (16.3-19.7)
Jan-23	2.0 (1.8-2.1)	14.9 (13.8-16.0)	11.7 (10.5-13.2)	17.7 (16.1-19.5)
Feb-23	2.0 (1.8-2.1)	14.7 (13.7-15.9)	11.7 (10.4-13.2)	17.5 (15.9-19.3)
Mar-23	1.9 (1.8-2.1)	14.6 (13.5-15.8)	11.7 (10.3-13.2)	17.3 (15.6-19.2)
Apr-23	1.9 (1.8-2.1)	14.5 (13.3-15.8)	11.6 (10.2-13.3)	17.1 (15.3-19.2)
May-23	1.9 (1.7-2.1)	14.4 (13.1-15.8)	11.6 (10.0-13.4)	16.9 (14.9-19.1)
Jun-23	1.9 (1.7-2.1)	14.3 (12.9-15.8)	11.6 (9.9-13.6)	16.7 (14.6-19.1)
Jul-23	1.9 (1.7-2.2)	14.2 (12.7-15.9)	11.6 (9.7-13.8)	16.5 (14.2-19.2)
Aug-23	1.9 (1.6-2.2)	14.1 (12.4-15.9)	11.6 (9.5-14.0)	16.3 (13.7-19.2)
Sep-23	1.9 (1.6-2.2)	14.0 (12.1-16.0)	11.5 (9.3-14.2)	16.1 (13.3-19.2)
Oct-23	1.9 (1.6-2.2)	13.8 (11.9-16.1)	11.5 (9.1-14.5)	15.9 (12.9-19.3)

Abbreviations: CI, confidence interval; RYO, roll-your-own.

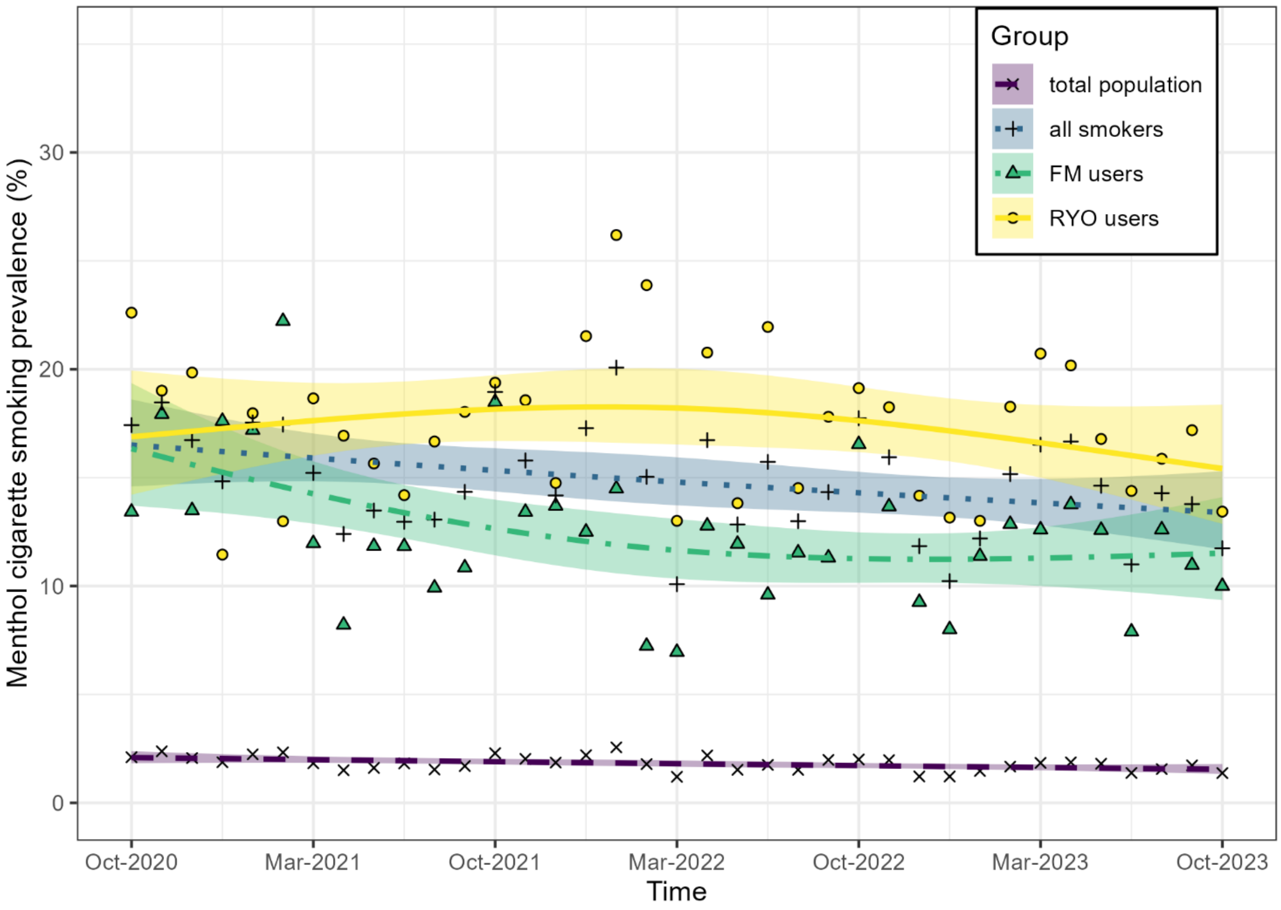

Figure S5: Unweighted modelled menthol cigarette smoking prevalence among the adult population ($N_{unweighted}$=81,293), all who smoke cigarettes ($n_{unweighted}$=9883), those predominantly smoking FM cigarettes ($n_{unweighted}$=5146), and those predominantly smoking RYO cigarettes ($n_{unweighted}$=4737) between October 2020 and October 2023. Shaded areas indicate 95% confidence intervals and dots show unmodelled estimates. Abbreviations: FM, factory-made; RYO, roll-your-own.

Table S9: Unweighted modelled estimates in menthol cigarette smoking prevalence among the adult population ($N_{unweighted}$=81,293), all who smoke cigarettes ($n_{unweighted}$=9883), and those predominantly smoking RYO cigarettes ($n_{unweighted}$=4737) between October 2020 and October 2023.

**Month-year**	**Menthol cigarette smoking, % (95% CI)**			
	Total population	All smokers	FM users	RYO users
Oct-20	2.1 (1.8-2.4)	16.5 (14.6-18.6)	16.3 (14.2-19.9)	16.9 (14.2-19.9)
Nov-20	2.1 (1.8-2.3)	16.4 (14.7-18.3)	16.0 (14.5-19.8)	17.0 (14.5-19.8)
Dec-20	2.1 (1.8-2.3)	16.3 (14.7-18.0)	15.6 (14.9-19.7)	17.1 (14.9-19.7)
Jan-21	2.0 (1.8-2.2)	16.2 (14.8-17.7)	15.2 (15.2-19.6)	17.3 (15.2-19.6)
Feb-21	2.0 (1.9-2.2)	16.1 (14.8-17.5)	14.9 (15.5-19.5)	17.4 (15.5-19.5)
Mar-21	2.0 (1.9-2.2)	16.0 (14.8-17.2)	14.6 (15.8-19.4)	17.5 (15.8-19.4)
Apr-21	2.0 (1.8-2.1)	15.9 (14.8-17.0)	14.3 (16.0-19.4)	17.6 (16.0-19.4)

Month-year	Menthol cigarette smoking, % (95% CI)			
	Total population	All smokers	FM users	RYO users
May-21	2.0 (1.8-2.1)	15.8 (14.8-16.9)	13.9 (16.2-19.4)	17.7 (16.2-19.4)
Jun-21	2.0 (1.8-2.1)	15.7 (14.8-16.7)	13.7 (16.4-19.4)	17.8 (16.4-19.4)
Jul-21	1.9 (1.8-2.1)	15.6 (14.7-16.6)	13.4 (16.5-19.4)	17.9 (16.5-19.4)
Aug-21	1.9 (1.8-2.1)	15.5 (14.6-16.5)	13.1 (16.6-19.5)	18.0 (16.6-19.5)
Sep-21	1.9 (1.8-2.0)	15.4 (14.5-16.4)	12.9 (16.7-19.6)	18.1 (16.7-19.6)
Oct-21	1.9 (1.8-2.0)	15.3 (14.4-16.4)	12.6 (16.7-19.7)	18.2 (16.7-19.7)
Nov-21	1.9 (1.8-2.0)	15.2 (14.2-16.3)	12.4 (16.7-19.8)	18.2 (16.7-19.8)
Dec-21	1.9 (1.7-2.0)	15.1 (14.1-16.2)	12.2 (16.7-19.9)	18.2 (16.7-19.9)
Jan-22	1.9 (1.7-2.0)	15.1 (14.0-16.2)	12.1 (16.6-20.0)	18.3 (16.6-20.0)
Feb-22	1.8 (1.7-2.0)	15.0 (13.9-16.1)	11.9 (16.6-20.0)	18.3 (16.6-20.0)
Mar-22	1.8 (1.7-2.0)	14.9 (13.8-16.0)	11.8 (16.6-20.1)	18.2 (16.6-20.1)
Apr-22	1.8 (1.7-2.0)	14.8 (13.7-15.9)	11.6 (16.5-20.0)	18.2 (16.5-20.0)
May-22	1.8 (1.7-1.9)	14.7 (13.7-15.8)	11.5 (16.5-20.0)	18.2 (16.5-20.0)
Jun-22	1.8 (1.6-1.9)	14.6 (13.6-15.7)	11.5 (16.4-19.9)	18.1 (16.4-19.9)
Jul-22	1.8 (1.6-1.9)	14.5 (13.5-15.6)	11.4 (16.4-19.7)	18.0 (16.4-19.7)
Aug-22	1.7 (1.6-1.9)	14.5 (13.5-15.5)	11.3 (16.3-19.6)	17.9 (16.3-19.6)
Sep-22	1.7 (1.6-1.9)	14.4 (13.4-15.4)	11.3 (16.3-19.4)	17.8 (16.3-19.4)
Oct-22	1.7 (1.6-1.8)	14.3 (13.4-15.3)	11.3 (16.2-19.2)	17.6 (16.2-19.2)
Nov-22	1.7 (1.6-1.8)	14.2 (13.3-15.2)	11.2 (16.1-19.0)	17.5 (16.1-19.0)
Dec-22	1.7 (1.6-1.8)	14.1 (13.3-15.1)	11.2 (15.9-18.8)	17.3 (15.9-18.8)
Jan-23	1.7 (1.6-1.8)	14.1 (13.2-15.0)	11.2 (15.8-18.7)	17.2 (15.8-18.7)
Feb-23	1.7 (1.5-1.8)	14.0 (13.1-15.0)	11.2 (15.5-18.5)	17.0 (15.5-18.5)
Mar-23	1.6 (1.5-1.8)	13.9 (12.9-14.9)	11.3 (15.3-18.4)	16.8 (15.3-18.4)
Apr-23	1.6 (1.5-1.8)	13.8 (12.8-14.9)	11.3 (15.0-18.3)	16.6 (15.0-18.3)
May-23	1.6 (1.5-1.8)	13.8 (12.6-15.0)	11.3 (14.7-18.3)	16.4 (14.7-18.3)
Jun-23	1.6 (1.4-1.8)	13.7 (12.5-15.0)	11.3 (14.3-18.3)	16.2 (14.3-18.3)
Jul-23	1.6 (1.4-1.8)	13.6 (12.3-15.1)	11.4 (14.0-18.3)	16.0 (14.0-18.3)
Aug-23	1.6 (1.4-1.8)	13.5 (12.1-15.1)	11.4 (13.6-18.3)	15.8 (13.6-18.3)
Sep-23	1.6 (1.4-1.8)	13.5 (11.9-15.2)	11.5 (13.2-18.3)	15.6 (13.2-18.3)
Oct-23	1.5 (1.3-1.8)	13.4 (11.7-15.3)	11.5 (12.9-18.4)	15.4 (12.9-18.4)

Abbreviations: CI, confidence interval; FM, factory-made; RYO, roll-your-own.

6. References

1. European Commission. Directive 2014/40/EU of the European Parliament and of the Council. In: European Commission, editor. Official Journal of the European Union2014.

2. European Commission. Revision of the Tobacco Products Directive. Internet. European Commission. Accessed 26 September, 2022. https://health.ec.europa.eu/tobacco/product-regulation/implementing-tobacco-products-directive-directive-201440eu/revision-tobacco-products-directive_en

3. Department for Health and Social Care. The Tobacco Products and Nicotine Inhaling Products (Amendment) (EU Exit) Regulations 2020. In: Department for Health and Social Care, editor. London, United Kingdom: UK Government; 2020.

4. Talhout R, van de Nobelen S, Kienhuis AS. An inventory of methods suitable to assess additive-induced characterising flavours of tobacco products. *Drug and Alcohol Dependence*. 2016/04/01/ 2016;161:9-14. doi:10.1016/j.drugalcdep.2015.12.019

5. Paschke M, Tkachenko A, Ackermann K, Hutzler C, Henkler F, Luch A. Activation of the cold-receptor TRPM8 by low levels of menthol in tobacco products. *Toxicology Letters*. 2017/04/05/ 2017;271:50-57. doi:10.1016/j.toxlet.2017.02.020

6. Hiscock R, Silver K, Zatoński M, Gilmore AB. Tobacco industry tactics to circumvent and undermine the menthol cigarette ban in the UK. *Tobacco Control*. 2020;29(e1):e138-e142. doi:10.1136/tobaccocontrol-2020-055769

7. Branston JR, Hiscock R, Silver K, Arnott D, Gilmore AB. Cigarette-like cigarillo introduced to bypass taxation, standardised packaging, minimum pack sizes, and menthol ban in the UK. *Tobacco Control*. 2021;30(6):708-711. doi:10.1136/tobaccocontrol-2020-055700

8. Advertising Standards Authority. Tobacco, rolling papers and filters. Advertising Standards Authority,. Updated 16 October 2013. Accessed 13 June, 2024. https://www.asa.org.uk/advice-online/tobacco-rolling-papers-and-filters.html

9. Conway L. *Prohibition of tobacco displays*. 2020:1-12. *Briefing paper*. Accessed 29 September 2022. https://researchbriefings.files.parliament.uk/documents/SN05537/SN05537.pdf

10. Haw S, Currie D, Eadie D, et al. Public Health Research. *The impact of the point-of-sale tobacco display ban on young people in Scotland: before-and-after study*. NIHR Journals Library; 2020.
